# Effect of food insecurity on the cognitive problems among elderly in India

**DOI:** 10.1186/s12877-021-02689-7

**Published:** 2021-12-18

**Authors:** Shubham Kumar, Anjali Bansal, Neha Shri, Nayan Jyoti Nath, Divya Dosaya

**Affiliations:** 1 International Institute for Population Sciences, Govandi East, Mumbai, 400088 India; 2 CHRIST (Deemed-to-be-University), Central Campus, Bengaluru, India; 3 Birla Institute of Technology and Science, Pilani, India

**Keywords:** Disability, ADL, IADL, India, Nutrition

## Abstract

**Background:**

Food Insecurity (FI) is a crucial social determinant of health, independent of other socioeconomic factors, as inadequate food resources create a threat to physical and mental health especially among older person. The present study explores the associations between FI and cognitive ability among the aged population in India.

**Methods:**

To measure the cognitive functioning we have used two proxies, word recall and computational problem. Descriptive analysis and multivariable logistic regression was used to understand the prevalence of word recall and computational problem by food security and some selected sociodemographic parameters. All the results were reported at 95% confidence interval.

**Results:**

We have used the data from the first wave of longitudinal ageing study of India (LASI), with a sample of 31,464 older persons 60 years and above. The study identified that 17 and 5% of the older population in India experiencing computational and word recall problem, respectively. It was found that respondents from food secure households were 14% less likely to have word recall problems [AOR:0.86, 95% CI:0.31–0.98], and 55% likely to have computational problems [AOR:0.45, 95% CI:0.29–0.70]. We also found poor cognitive functioning among those experiencing disability, severe ADL, and IADL. Further, factors such as age, education, marital status, working status, health related factors were the major contributors to the cognitive functioning in older adults.

**Conclusion:**

This study suggest that food insecurity is associated with a lower level of cognition among the elderly in India, which highlight the need of food policy and interventional strategies to address food insecurity, especially among the individuals belonging to lower wealth quintiles. Furthermore, increasing the coverage of food distribution may also help to decrease the burden of disease for the at most risk population. Also, there is a need for specific programs and policies that improve the availability of nutritious food among elderly.

## Introduction

In India, with the rapid increase in life expectancy, the age pyramid structure shifted to the older population. In 2019, around globe, there were 703 million older persons aged 65 years or above which further estimated to exorbitantly double up over the next three decades up to 1.5 billion in 2050 [1]. The World Population Ageing Report, 2019, added that the rise in ageing population would be significantly high in the Eastern and South-eastern regions of Asia. Among the identified regions, low- and middle-income countries like India, downtrodden with multiple SDHs, population ageing will further have adverse consequences on social and economic conditions [2, 3]. Ageing is a global phenomenon, and it is often viewed and commemorated as outcome of better public healthcare system, and coup over the Social Determinants of Health (SDH). The public health consequence of ageing group simultaneously pose threat to mental health, particular the cognitive ability [4, 5].

Despite India being the fastest growing economy globally, the country ranked at 94 out of 107 countries on Global Hunger Index, much behind Bangladesh, Pakistan, and Nepal, with hunger levels categorized as ‘serious’ [6]. Food insecurity possess a very big challenge in front of the policy makers, which sometimes hinders the development of cognitive functioning among children, and in later ages [7–12]. Food insecurity (FI) is “limited or uncertain availability of nutritionally adequate and safe foods or limited or uncertain ability to acquire acceptable foods in socially acceptable ways” [13]. Studies have also defined FI from an economic perspective as “disruption of food intake or eating patterns because of lack of money and other resources” [14]. In 2020, approximately 17.2% of the global population, or 1.3 billion people, have experienced food insecurity at moderate levels where access to sufficient food is hindered due to numerous social determinants of health [15]. The latest Longitudinal Ageing Study in India (LASI) reported that 6% of older adults age 45+ had reduced the size of means, 5% were hungry due to inadequate food, and 4% did not eat for a full day as the food is unavailable in the past 12 months. Madhya Pradesh (13.6%), Bihar (10.4%), and Jharkhand (10.1%) significantly reported constraints in food availability, much higher than the national average (8.1%) [15]. The access to moderate food insecurity may not necessarily be hunger but certainly at the greater risk of malnutrition and poor health (ibid.). A more significant proportion of food insecurity exists in low- and middle-income countries, creating a threat to developing many chronic diseases [16]. Food insecurity is one of the variables influencing the severe state of hunger across the Indian population. FI is a crucial social determinant of health, independent of other socioeconomic factors [7], as inadequate food resources create a threat to physical and mental health of people of all ages [8].

Aging cause several changes in the physiological functions, including cognitive functioning, bone loss, etc. Multiple works of literature have shown that the FI is associated with several chronic physical health conditions [17–19]. Studies found that the lack of inadequate food leads to development of various chronic health condition such as hypertension [20], cardiovascular diseases [21–23], and diabetes [21]. Other than physical health, food insecurity has been known to impact cognitive function and mental health [11, 12, 24]. Inadequate nutrition caused by FI has also shown an association with unfavourable health outcomes such as obesity, anxiety, depression, and suicide, which activates the hypothalamic-pituitary-adrenal axis and triggers the secretion of glucocorticoids in the adrenal glands [24, 25]. The deficiency of several vitamins and micronutrients also leads to various cognitive impairment which often leads to chronic health state, like Alzheimer’s [23, 26–28]. So it is important to study the relevance of food insecurity in context of elderly which leads to cognitive impairment. Longitudinal and cross-sectional studies conducted in the western part of the world concluded that aging is a primary factor for cognitive decline and cognitive impairment [9, 12]. Studies conducted in 2004 among the adult population from Puerto Rican, Boston, USA, stated a stronger association between food insecurity and cognitive function in older participants than the younger ones [24]. At the same time, another study conducted from 2011 to 14 by National Center for Health Statistics (USA) found that food insecurity is inversely associated with cognitive function among older adults, which translates into a higher risk of cognitive impairment over time [9].

Existing studies have established the strong association of food insecurity with children’s cognitive functioning [10, 29]. However, little is known about whether food security is associated with working memory, semantic memory, and behavioural issues among the elderly aged population in India. Several other studies establish a significant impact of FI on cognitive functioning, highlighting the importance of food policy and reducing disease burden through alleviating FI. However, there is a lack of adequate evidence to establish the association between FI and cognitive ability among the aged population, 60+ in India. Given the sparse literature in the Indian context, the objective of the present study was to examine whether the prevalence of food insecurity has an association with cognition ability among the aged population above 60 years.

## Materials and methods

### Data

We have used the data from the first wave of longitudinal ageing study of India (LASI), which has a carried out for all 35 states (exclude Sikkim) and union territories (UTs) of elderly aged 45 years and above.

LASI is India’s first-ever survey which provides comprehensive information on demographics, household economic status, chronic health conditions, symptom-based health conditions, functional health, mental health (cognition and depression), biomarkers, health insurance and healthcare utilization, family and social networks, social welfare programmes, work and employment, retirement, satisfaction, and life expectations. The survey has well-developed tools to evaluate the effect of changing policies on the health outcomes among older adults in India.

LASI wave one was supported by the Ministry of Health and Family Welfare (MoHFW), the Government of India, the National Institute on Aging (NIA), and the United Nations Population Fund, India (UNFPA). LASI is a collaborative study of three nodal agencies: International Institute for Population Sciences (IIPS), Harvard T.H. Chan School of Public Health (HSPH), and University of Southern California (USC) and several other national and international institutions.

The LASI has used a multistage stratified area probability cluster sampling to achieve a nationally representative sample of older adults. This stage sampling design has adopted rural areas and a four-stage sampling design for urban areas across the states and UTs. In the first stage of sampling, primary sampling units (PSUs) were selected, which were Tehsils and Talukas. In the second stage, villages and wards were selected for rural and urban areas respectively in selected PSUs. The third stage involved selection of households in rural areas and census enumeration blocks (CEBs) in urban areas. In the fourth stage, households were selected from CEBs in urban areas [15].

### Study variables

#### Outcome variables

The outcome variable for this study is cognition. In addition to that, working memory (word recall and computational) is considered a proxy measure of cognition [30]. In the LASI questionnaire, the word recall problem has been measured using three sets of list, list1, list2, and list3, which consists of 10 words in each. Respondents were asked to recall as many words as they can, and were categorised as '0' if they did not recalled any of the words from the lists and 1 otherwise.

Similarly, the computational problem has been assessed based on two numerical questions asked in the survey. Further, the answer has recorded either ‘correct’ or ‘incorrect’ and recoded in ‘yes’ and ‘no’.

#### Response variables

The response variables for this study are food insecurity (severe, moderate and secure); gender (male and female); age (60–69 and 70 years and above); marital status (currently married, never married, divorced/separated/eserted/widowhood), education (no education, below primary, primary, secondary, and higher); living arrangements (living alone, with spouse and with others); place of residence (rural and urban); wealth index (poorest, poorer, middle, richer and richest); currently working (yes and no); self-rated health (poor and good; physical activity (yes and no); tobacco use (no and yes); ADL disability (severe, moderate and no disability), and IADL disability (severe, moderate and no disability).

#### Computation of food insecurity, IADL and ADL disability scale

In our study, food insecurity was measured using five questions, All the questions were asked as period reference of the last 12 months, The questions were, did you ever reduce the size of your meal? Did you eat enough food of your choice? Were you hungry but didn’t eat because there was not enough food in your household? Did you ever not eat for a whole day because there was not enough food in your household?, and Do you think you have lost weight in the last 12 months because there was not enough food in your household? All the responses were coded as yes and no, and were further categorised into three categories severe, moderate and secure. The methodology for the computation of food insecurity scale was developed using previous studies [31–33].

Activities of daily living (ADL) and Instrumental activities of daily living (IADL) disability constructed from five (bathing, dressing, mobility, feeding, and toileting) and seven (preparing a hot meal (cooking and serving), shopping for groceries, making telephone calls, taking medications, doing work around the house or garden, managing money, such as paying bills and keeping track of expenses and getting around or finding an address in an unfamiliar place) activities. The methodology for computation of the IADL scale was adopted by Katz (1963), in which they used eight indicators to measure the scale, [34], but we used six indicators based on the availability in LASI. Similarly the ADL disability scale was adopted from Lawton (1969) [35], where they have used six indicators to measure ADL, but we used five indictors subject to availability in the LASI study. Both the ADL and IADL disability was categorized into the three categories as “severe”, “moderate”, and “no disability”.

### Statistical measures

Descriptive analyses were used to understand the prevalence of word recall and computational problem by food security and some selected sociodemographic parameters. To estimate the odds ratio, binary logistic regression was performed to determine the adjusted association between cognition problems and food security and sociodemographic parameters. The equation of binary logistic regression can be written as follow:

$$\log \left(\frac{p_i}{1-{p}_i}\right)={\beta}_{0+{\beta}_1{x}_1+{\beta}_2{x}_2}\dots {\beta}_k{x}_k$$

Where p is the probability, *β*_0_ is the intercept, *β*_1_, *β*_2_ to *β*_*k*_ are the coefficients and *x*_1_ *x*_2_ to *x*_*k*_ are the independent variables. All the analyses have been performed using STATA version 16, and results are reported at 5% level of significance.

## Results

The first wave of LASI took place in 2016–17 with a national sample of 72,250 older adults aged 45 and above, including 31,464 elderly age 60 and above and 6749 oldest-old persons age 75 and above. However, our study is concerned with 31,464 elderlies from 60 years and above.

**Fig. 1:**
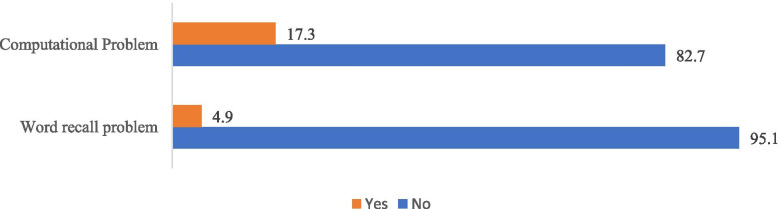
Proportion of working memory (word recall and computation) among elderly

**Fig. 2:**
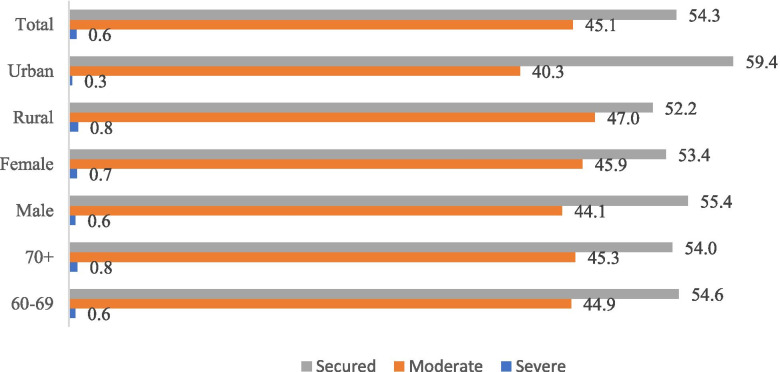
Food insecurity among elderly by age, gender and place of residence

It was found that 17.3% of the participants had word recall working memory problem and almost 5% had computational working memory problems. The socio-economic characteristics and health status of the study participants are shown in Table 1. Word recall problem and computational problems was found to be highest among respondents from food insecure households and adults aged 70 and above. Thirty nine percent of the respondents from severely food insecure households had computational problems while 7.3% have word recall problems. Higher proportion of females has word recall problems and computational problems. Around 10% of male have computational problem whereas 23.8% of female had computational problem. This gender difference was not much pronounced in case of word recall problems. Age-wise, both of this cognitive impairment problem was found to be highest among respondents aged 70 and above and around 8 and 24% of them have had word recall problem and computational problems. As far as marital status is concerned, working memory problems was lowest among currently married respondents. Roughly one-fourth of the respondents without any education had computational problems and word recall problem was found to be 5.8% among respondents without any education. However, the proportion of respondents with working memory problem decreased with the higher levels of education. The working memory problem was highest among respondents living with person other than spouse followed by respondents living alone and least among respondents living with spouse. Surprisingly, word recall problem was highest (5.6%) among respondents from rural areas. However, among urban resident’s word recall problem was found to be 4.7%. In contrast, 19.1% of the rural residents have computational memory problem whereas among urbanites it was found to be 13.2%. As we move from poorer to richer wealth quintile, the proportion of respondents with working memory problems decline substantially. For instance, 24.1% of the respondents from poorest wealth quintile have computational problems whereas among respondents belonging to richest wealth quintile it was 12.7%. Similarly, around 5% of respondents from poorest wealth quintile have word recall problems and 4.3% of richest individuals have word recall problems. Word recall problem was thrice higher among respondents who were not currently working than currently working respondents (2.4%). A slightly higher proportion (18.5%) of currently not working respondents have computational problem whereas computational problem was found to be only 11.6% among currently working respondents. Surprisingly, one-fifth (21.1) of the respondents who reported their health to poor had computational problems. About 4.3 and 2.1% of respondents who reported their health to be poor and good respectively had word recall problems. Surprisingly, tobacco users had lower levels of computational and word recall problems in compared to non-tobacco users. Around half of the respondents with severe ADL disability had computational problems whereas it was prevalent among 14% respondents without any ADL disability. Similarly, 29% of the respondents who had severe ADL disability had word recall problems. People with severe ADL or IADL disability had highest computational and word recall problems. Analogously, 46% of respondents with IADL disability were found to have computational problems. However, word recall memory was found to be 4 and 2.3% respectively for those who had moderate and no IADL disability (Figs. 1 and 2).

**Table 1:** proportion of working memory (word recall and computation) problems among elderly by food insecurity and selected sociodemographic parameters

	Word-recall problem	Computational problem	Total
	%	%	N
**Food Insecurity**			
Severe	7.3	29.5	199
Moderate	4.4	18.8	14,178
Secure	5.4	16.0	17,088
**Gender**			
Male	4.7	10.2	14,931
Female	5.1	23.8	16,533
**Age**			
60–69	2.8	12.6	18,410
70+	7.9	24.0	13,054
**Marital Status**			
Currently married	4.1	12.8	19,536
never married	7.9	20.9	225
divorced/separated/deserted	6.3	24.8	11,703
**Education**			
No Education	5.8	25.9	17,782
Below primary	3.6	9.0	3598
Primary	3.3	6.0	3520
Secondary	4.6	5.4	5285
Higher	1.9	2.2	1278
**Living arrangements**			
Living alone	4.8	21.7	1787
With spouse	3.9	12.7	19,176
With others	6.8	25.1	10,501
**Place of residence**			
Rural	4.7	19.1	22,196
Urban	5.6	13.2	9268
**Wealth quintile**			
Poorest	5.0	24.1	6829
Poorer	5.0	18.7	6831
Middle	6.2	16.2	6590
Richer	4.0	13.5	6038
Richest	4.3	12.7	5175
**Currently working**			
Yes	2.4	11.6	9483
No	6.6	18.5	13,197
**Self-rated health**			
Poor	4.3	21.1	4630
Good	2.1	14.1	26,181
**Use of tobacco**			
No	4.2	17.5	18,665
Yes	3.7	14.9	12,539
**ADL disability**			
Severe ADL	29.0	50.6	999
Moderate ADL	6.0	20.7	6045
No ADL	2.7	14.3	24,291
**IADL disability**			
Severe IADL	22.8	46.0	1859
Moderate IADL	4.0	18.6	13,281
No IADL	2.3	11.8	16,164

Table 2 shows the results obtained from the logistic regression analysis of socio-economic status and health status which determine the working memory problems among the older adults. Respondents from food secure households were 29% less likely to have word recall problems [AOR:0.86, 95% CI:0.31–0.98] than respondents from severely food insecure households. Further, with the increasing household food security the prevalence of computational problem declined significantly. Females were 2.75 times more likely than men to have computational problem [AOR:2.75, 95% CI:2.27–3.32]. Unsurprisingly, respondents aged 70 and above were 2.93 odd times and 2.18 odd times more likely to have word recall problems and computational problems respectively [AOR:2.93, 95% CI:2.16–3.97; AOR:2.24, 95% CI:1.94–2.59]. The likelihood of having word recall problem was significantly higher among never married and divorced/separated/deserted respondents than currently married respondents. For instance, never married respondents were twice more likely to have word recall problems than married respondents [AOR:02.03, 95% CI:1.02–4.02]. Computational problem was significantly inversely associated with the higher levels of education and place of residence. Respondents who had the higher level of education were 94% less likely to have computational problem [AOR:0.06, 95% CI:0.04–0.10] than those who respondents who were not educated. Respondents who were living with people other than spouse were at higher risk of having word recall and computational problem. As evident, respondents living with people other than spouse were 43 times more likely to have word recall problem [AOR:1.43, 95% CI:0.98–2.10] and 21% more likely to have computational problem [AOR:1.21, 95% CI:0.99–1.46] than those living alone. However, respondents living with spouse were less likely to have these cognitive issues than respondents living alone though insignificant in case of word recall problem. As we move from lower to higher wealth quintiles, the likelihood of having computational problems decreased significantly. Respondents from richest wealth quintile were 54% [AOR:0.45, 95% CI:0.38–0.54] less likely to have computational cognitive impairment than respondents from poorest wealth quintile. Working status was found to be significantly inversely associated with cognitive issues. Respondents who were not working currently were 2.85 odd times [AOR:2.85, 95% CI:1.94–4.19] more likely to have word recall problem compared to currently working respondents. Similarly, not currently working respondents were 1.73 odd times [AOR:1.73, 95% CI:1.47–2.04] more likely to have computational problems in comparison to respondents who were not working. Respondents who rated their health as good were 53% [AOR:0.47, 95% CI:0.36–0.62] and 39% [AOR:0.61, 95% CI:0.73–0.91] significantly less likely to have word recall problems and computational problem respectively. Use of tobacco was found to be significantly negatively associated with computational problems among older adults. The likelihood of having word recall problem and computational problem was significantly lower among those who had moderate difficulty with ADL activities than those who had severe difficulty with ADL activities [AOR:0.15, 95% CI:0.11–0.21; AOR:0.25, 95% CI:0.20–0.32]. further, respondents without any ADL difficulty were 94% [AOR:0.06, 95% CI:0.05–0.09] less likely to have word recall problem and 86% [AOR:0.16, 95% CI:0.13–0.20] less likely to have computational problem than respondents with severe ADL disability. A significant positive association exists between IADL disability and reduced cognitive impairment. Respondents without any IADL disability were 93% [AOR:0.07, 95% CI:0.11–0.10] less likely to have word recall problem than those who did not have any difficulty with ADL activities.

**Table 2:** Logistic regression of working memory (word recall and computation) by food security and selected sociodemographic parameters

	Word recall problem		Computational problem	
Food Insecurity	Odds ratio	CI at 95%	Odds ratio	CI at 95%
Severe				
Moderate	0.58**	0.26–1.27	0.55**	0.35–0.84
Secure	0.86*	0.31–0.98	0.45***	0.29–0.70
**Gender**				
Male				
Female	1.08	0.74–1.57	2.75***	2.27–3.32
**Age**				
60–69				
70+	2.93***	2.16–3.97	2.18***	1.91–2.49
**Marital Status**				
Currently married				
never married	2.03**	1.02–4.02	1.80**	1.18–2.74
divorced/separated/deserted	1.60**	1.12–2.27	2.24***	1.94–2.59
**Education**				
No Education				
Below primary	0.60**	0.39–0.92	0.28***	0.22–0.35
Primary	0.59***	0.38–0.80	0.18***	0.14–0.23
Secondary	0.78	0.29–2.06	0.16***	0.07–0.37
Higher	0.30***	0.19–0.48	0.06***	0.04–0.10
**Living arrangements**				
Living alone				
With spouse	0.80	0.49–1.28	0.52***	0.42–0.64
With others	1.43**	0.98–2.10	1.21**	0.99–1.46
**Place of residence**				
Rural				
Urban	1.20	0.73–1.98	0.64***	0.50–0.81
**Wealth quintile**				
Poorest				
Poorer	0.99	0.72–1.37	0.72***	0.62–0.83
Middle	1.25	0.68–2.31	0.60***	0.46–0.78
Richer	0.78	0.56–1.07	0.49***	0.40–0.59
Richest	0.85**	0.67–1.10	0.45***	0.38–0.54
**Currently working**				
Yes				
No	2.85***	1.94–4.19	1.73***	1.47–2.04
**Self-rated health**				
Poor				
Good	0.47***	0.36–0.62	0.61***	0.54–0.69
**Use of tobacco**				
Yes				
No	0.86	0.70–1.07	0.82***	0.73–0.91
**ADL disability**				
Severe ADL				
Moderate ADL	0.15***	0.11–0.21	0.25***	0.20–0.32
No ADL	0.06***	0.05–0.09	0.16***	0.13–0.20
**IADL disability**				
Severe IADL				
Moderate IADL	0.13***	0.11–0.17	0.26***	0.22–0.31
No IADL	0.07***	0.05–0.10	0.15***	0.13–0.18

*p < 0.1, **p < 0.05, ***p < 0.000

## Discussion

In this study, we investigated the relationship between food insecurity and cognitive functioning among elderly aged 60+ using the LASI data. Here we used two indicators to measure cognitive functioning, word recall and computational problem. The study identified that 17 and 5% of the older population in India were experiencing computational and word recall problem, respectively. Our study found that socio-economic Status (SES) and food insecurity were significant in explaining the cognitive functioning among older adults in India (60+).

In our study we hypothesized that those who are food insecure were more likely to acquire cognitive impairment, and our results proved our hypothesis. It was found that food secure elderly were 0.71 and 0.45 odd times less likely to have word recall problem and computational problem respectively than who were insecure. This finding is alarming since accelerated decline in cognitive functioning have been consistently reported for older aged adults with food insecurity [9, 12, 24, 25, 36–39]. Previous research has found that food -insecure adults tend to have lower intake of various vitamins and food groups [9]. Consuming a healthy diet has always lower the risk of developing a poor mental health [40, 41], decline in adults cognitive functioning [12, 36]. A longitudinal cohort study**,** also found the association of very low food security with the lower cognition impairment in a two-year-long span [24]. In addition, the study found that the differences in global cognitive scores among the participants with low food security and those with food security were equivalent to accelerating cognitive ageing by 8 years [36]. Also, cross-sectional studies reported the relationship between food insecurity and cognitive functioning. A study by Gao et al. (2009) and Tong et al. (2018) had reported that the odds of cognitive impairment increased to two manifolds if the individuals are living with very low food security (VLFS) compared to those who are secure [24, 38]. The accelerated cognitive aging associated with food insecurity can lead to poor mental health which can result in acquiring functioning disability at the later ages. The studies based on micronutrient deficiency also found cognitive impairment among those who have deficiency of Vitamin B [26, 27]. Also, the findings of our study corroborates with the findings from the latest systematic review done by Iolascon et al., 2017, where they found that availability of 16 major micronutrients in the diets can reduce the risk of cognitive impairment among elderly in world [19].

We also investigated the cognitive functioning w.r.t SES which can be interlinked with mental health situation especially among older adults in India. We found low cognitive functioning among the older adults (70+), as the ability to think and recall diminish over time. Similar to our findings, Pliatsikas et al. (2019) and Fournet et al. (2012) also showed that working memory declines with age [42, 43], which can often leads to deficits such as Alzheimer’s, Parkinson’s disease, and aphasia [44, 45]. We also found that the educational level of individual also impacts the cognitive functioning. We found that increasing educational decreases the odds of both word recall problem and computational problem. Consistent to our findings a study conducted in Taiwan found a positive association of educational with working memory [43]. The educational status strengthened the long term memory representations [46], which increases the working memory in older ages, which can delay the onset of various mental health disease. The wealth quintile also found to be a significant predictor in assessing the cognitive functioning among the elderly. Consistent with our findings, one study based on Latino respondents found that the wealth quintile of the household significantly contributes to cognitive functioning [47].

Furthermore, we found that poor cognitive functioning among those experiencing disability, severe ADL, and IADL. For example, those who have no ADL disability were 0.06 odd times less likely to have a word recall problem and 0.16 odd times less likely to have a computational problem. Similar to our findings, a longitudinal study based on U. S adults found that for those with the lowest level of cognition, the odds of getting a disability in the older ages increases to 1.58 times [48]. Another study by Gateau and Fabrigoule (1997) also found that those with low cognitive performance had a higher risk of disability at later ages [49].

## Conclusion

Our findings from this study suggest that food insecurity is associated with a lower level of cognition among the elderly in India. These findings highlight the importance of food policy and interventional strategies to address food insecurity, especially among the individuals belonging to lower wealth quintiles. Furthermore, increasing the coverage of food distribution may also help to decrease the burden of disease for the at most risk population, which further increases the healthy life expectancy among the aged population. So, there is a need for specific programs and policies that improve the availability of nutritious food, which help alleviate food insecurity and improve cognitive mental health among the vulnerable population.

## Data Availability

The datasets analysed during the current study are from Longitudinal Ageing Survey of India (LASI). The data is freely available from the Gateway to Global Aging Data.
